# Examining socio-cognitive factors and beliefs about mindful eating in healthy adults with differing practice experience: a cross-sectional study

**DOI:** 10.1186/s40359-022-00977-4

**Published:** 2022-11-15

**Authors:** Christian Erik Preissner, Anke Oenema, Hein de Vries

**Affiliations:** Department of Health Promotion, Maastricht University, 6200 MD Maastricht, The Netherlands

**Keywords:** Mindful-eating, I-change-model, Determinants, Motivation, Awareness

## Abstract

**Background:**

Mindful eating (ME), defined as a “non-judgmental awareness of bodily and emotional sensations regarding food consumption”, may be a promising strategy to promote healthy eating behaviors. However, little is known about the psychosocial factors and underlying beliefs that explain ME adoption.

**Methods:**

Participants (*N* = 282; *M*_age_ = 43.2) responded to an online questionnaire based on the I-Change Model. Groups with different frequencies of prior engagement in ME, i.e., low (*n* = 82; LME), medium (*n* = 96), and high (*n* = 104), were compared via (M)ANOVAs on factors and individual beliefs regarding predisposing (i.e., habits, experience with mindfulness, emotional eating, facets of ME), pre-motivational (i.e., knowledge, behavioral cognizance, risk perception, cues to action), and motivational factors (i.e., attitudes, self-efficacy, social influence) as well as their intentions and action planning. Bivariate correlations and a forward-stepwise regression with ICM constructs were conducted to examine model fit.

**Results:**

LME had a greater habit of mindless eating and significantly lower internal awareness, cognizance, cues, and less favorable attitudes, self-efficacy, engagement and support by their social environment, intention, and action plans about engaging in ME than the other two groups. Less habitual mindless eating, and greater experience, internal awareness, cognizance, susceptibility, support, and intention explained 54% of the variance in ME.

**Discussion and conclusion:**

Results indicate that individuals need to be treated differently when promoting ME with respect to their psychosocial characteristics, rather than as a single group with homogenous baseline beliefs, abilities, support, and motivation. Future longitudinal research should examine which determinants are predictors of ME to better tailor program contents.

**Supplementary Information:**

The online version contains supplementary material available at 10.1186/s40359-022-00977-4.

## Introduction

About half of the European adults have overweight or obesity [1]. Mindful eating (ME) behavior may be a promising approach to promote healthy lifestyle behaviors and prevent health implications associated with overweight and obesity. Mindfulness applied toward eating is described as a sustained attention to sensory components of food (e.g., smell, taste, texture) and a non-evaluative awareness of bodily and emotional sensations related to the eating experience [2, 3].

Systematic reviews and meta-analyses have indicated the promise of ME to address a range of obesity-related eating behaviors [4–6]. Specifically, previous studies found ME to mitigate various eating-related outcomes such as food cravings [7], overeating [8], and the consumption of energy-dense foods [9]. Earlier studies also found ME to promote fruit and vegetable intake [10], the perceived benefits of healthy snacking [11] as well as a slower pace of eating and feeling fuller on a smaller portion size [12].

Whereas mindfulness is often cultivated using formal exercises that are performed at a set time (e.g., audio-guided exercises or body scans) [4, 13], ME behavior constitutes an informally practiced mindfulness during everyday life, that is synchronized with a food-related situation or behavior [3]. Mindfulness-based programs encourage participants to frequently practice ME between sessions to promote sustained practice behavior after intervention completion [14, 15]. Like other health promoting behaviors, ME must be successfully integrated into an individual’s lifestyle to elicit meaningful, long-term health benefits. To ensure that participants are supported in making such sustainable changes to their eating behavior, it is crucial to identify and understand what factors are associated with the uptake of ME practice.

Earlier research has suggested the importance of motivation, intention, positive outcome expectations, and attitudes for the adoption of mindfulness practice behavior (see, e.g., the Liverpool Mindfulness Model; [16]). In addition, previous studies have identified attitudes [17, 18], habits [19] as well as action planning [20] as predictors of mindfulness practice. Social norms and both positive and negative outcome expectations were also found to predict mindfulness practice intentions [13]. Yet, there is a paucity of empirical work that provides an in-depth analysis of the relevant factors and beliefs associated with eating-related mindfulness, such as beliefs about its benefits, partaking for different motivational reasons or because of past engagement in related, transferable actions [21, 22]. Determinant studies using an appropriate theoretical framework are warranted to address this gap in the literature. This can inform the more tailored design and effect evaluation of ME interventions, that may otherwise favor individuals with an already established foundation for practicing new healthy eating behaviors.

### Theoretical backdrop

**Fig. 1:**
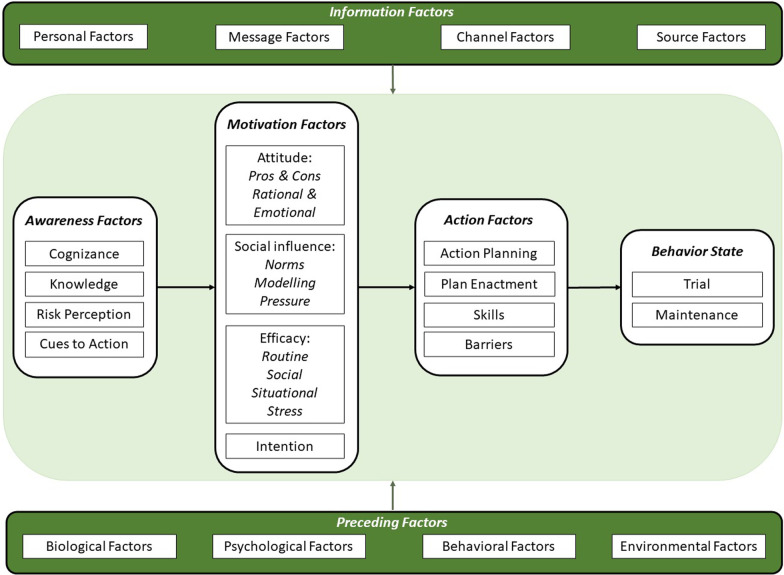
The I-Change Model [27]

To date, a number of behavior change theories have been utilized to understand and promote different healthy eating behaviors [23], including the Theory of Planned Behavior [24], Social Cognitive Theory [25], and the Trans Theoretical Model [26]. One model that integrates the ideas of such theories is the I-Change Model (ICM; [27]; see Fig. 1). The purpose of this model is to provide an integrative framework for explaining individuals’ engagement in health behaviors [27]. The ICM distinguishes between three stages of motivational change (pre-motivational, motivational, and post-motivational phase) and the awareness-, motivation-, and action-related factors determining the transition through these phases [28].

Within the pre-motivational phase, individuals have to become aware of cues regarding a health problem, develop knowledge about a problem, learn about the risks of a problem as well as reflect on and become cognizant of their own (risk) behaviors and corresponding level of risk. These factors may prompt an initial motivation for adopting a healthier behavior. Within the second, motivational phase, factors such as attitude beliefs (i.e., rational and emotional beliefs about the advantages and disadvantages of a behavior), social influence beliefs (norms, example behaviors of others, i.e., modeling, and social support), and self-efficacy become important as determinants of a person’s intention to adopt a healthier behavior. Third, post-motivational action factors may increase the chances of the positive intention being translated into the realized healthy behavior. These factors include a person developing preparatory actions to identify appropriate goals (goal setting), choosing appropriate goals (action plans), and strategies to maintain these plans in challenging situations (coping plan).

Whereas social cognitive models outline the most important factors for understanding and changing health behaviors, the belief structure determining these factors may vary in different populations [29–31]. It is therefore important to identify beliefs associated with awareness, motivation, and action to optimally guide intervention development [27].

The ICM has previously been applied to several health behaviors (see, e.g., [32–35]) including eating-related behaviors such as fruit and vegetable intake [36] and eating in moderation [37]. Therefore, this model may also present a suitable, comprehensive theoretical framework to guide an exploration of the beliefs underlying individuals’ decision to adopt ME.

### Purpose

The aim of this exploratory study was to identify relevant awareness-, motivation-, and action-related factors and beliefs associated with ME practice in healthy adults (i.e., individuals without eating disorder symptomatology, severe psychiatric disease, or chronic disease). To explore which beliefs are distinctive between those who do and do not already engage in ME behavior, we compared individuals with limited practice experience and those with intermediate and greater experience. Using the ICM as a backdrop, we first analyzed whether these groups differ on predisposing socio-demographic factors. Second, we compared the groups on awareness-, motivational-, and action-related beliefs. Third, we analyzed how well our model using ICM factors explains the variance in ME behavior. As this study constitutes exploratory, cross-sectional work, our primary focus was on comparisons between groups to gain insight into awareness-, motivation-, and action-related factors underlying ME as opposed to correlational and regression analyses. This greater understanding is essential for informing future interventions that encourage the adoption of ME for wider, non-clinical audiences [21, 38].

## Methods

### Participants

This cross-sectional study was conducted between April and June 2021 in Germany. In line with the aim of identifying beliefs, recruitment efforts targeted a variety of channels to obtain a sample with heterogeneity in their prior experience with mindfulness-based practices. Participants were recruited via advertisements posted in newsletters (e.g., that of community centers offering meditation or prayer groups), on social media, and through the online survey platform SurveyCircle [39]. The latter is an international volunteer-based research platform with a point-based incentive mechanism for study participation; additional compensation can be specified by the researcher. We provided participants with the option to participate in a raffle to win one of seven 15€ gift cards. Studies are advertised and open to the public on the SurveyCircle website as well as via their various social media channels (i.a., Twitter, Facebook, LinkedIn, Reddit). Participants were required to be proficient in the German language, currently reside in Germany, have adequate computer literacy to complete the online questionnaire, and be over the age of 18. Exclusion criteria were limitations that could influence appetite such as (i) currently undergoing treatment for chronic diseases such as cancer and (ii) being diagnosed with binge eating disorder, bulimia or anorexia nervosa, depression, or other severe psychiatric disease. A total of 282 individuals disclosed in the informed consent that they met the criteria for inclusion and completed the survey.^1^.

### Procedure

This study was conducted in accordance with the Declaration of Helsinki and approved by the FHML Research Ethics Committee at Maastricht University (#FHML/HEP_2021.547). Eligible individuals, based on the self-disclosure of the respective criteria by providing informed consent, were provided with information about the study (aims, procedure, benefits) and presented with an informed consent form explaining voluntary participation and anonymity, and the use of data. Individuals were asked to indicate their consent before being redirected to the questionnaire on Qualtrics. On average, the questionnaire took 20 min to complete. Participants were able to skip questions as well as save and resume their progress at any time. After reaching the end of the questionnaire, participants had the option to participate in the separately conducted raffle.

### Measures

The questionnaire assessed participants’ socio-demographic characteristics, facets of ME, and social-cognitive constructs of the ICM. Participants were provided with a brief introductory passage to each section that informed them about the respective construct and response options. A description of mindless eating and ME was presented in lay terms before participants were directed to the ICM constructs. These introductory passages and the descriptions can be obtained from the first author upon reasonable request. The questionnaire was piloted for comprehension with individuals from the target group and ambiguities were adjusted according to participants’ qualitative feedback. These individuals were not eligible for filling out the final questionnaire.

#### ICM preceding factors

##### Demographic variables

Participants were asked to indicate their age (years), gender (male = 0, female = 1, other = 2), highest level of formal education (low: none = 0, less than high school = 1; medium: high school = 2, vocational/trade school = 3; high: university/technical college = 4, doctorate = 5), current living situation (alone = 0, with others = 1), employment (employed full-time/part-time = 1, full-time/part-time student = 2, retired/early disability retirement = 3, other = 4) as well as their nationality (German = 1, else = 0) and place of birth (Germany = 1, else = 0).

##### Additional predisposing variables

On two single items, participants self-reported eating a specific diet due to food intolerances or food restrictions (no = 0, yes = 1) and their prior experience with mindfulness in general (never having heard of or never having attempted it = 1, some experience = 2, being (very) experienced = 3), respectively. Further, emotional eating was assessed by the emotional eating subscale of the validated short form of the Three Factor Eating Questionnaire [40, 41]. Three items assessed whether participants ate in response to being anxious, feeling blue, and feeling lonely (α = 0.80). All items were scored from 1 (*definitely false*) to 4 (*definitely true*).

##### Eating-related mindfulness skills

Participants preceding eating-related mindfulness skills were measured using the 29-item Four Facet Mindful Eating Scale (FFaMES; [42]. This measure has previously demonstrated good internal consistency for its subscales (Cronbach’s alphas ≥ 0.82; [42]). Individuals were asked to indicate their agreement with each statement from 1 (*never*) to 5 (*very often*). The scale assesses four sub-skills: non-reactance (i.e., maintaining a mental distance from immediate needs to eat; nine items; α = 0.90), non-judgment (i.e., accepting one’s eating behaviors without negative self-judgment; eight items; α = 0.90), external awareness (i.e., observing the effects of environmental factors on one’s eating; six items; α = 0.78), and internal awareness (i.e., observing the effects of internal processes on one’s eating; six items; α = 0.86) (see [42]). Items can be found in Additional file 1: Table 1. A German version of the FFaMES was developed by Carrière et al. [43] considering recommended forward-and back-translation procedures by independent, bilingual translators [44, 45]. In line with the authors’ recommendations, subscale scores rather than a summary score were calculated based on the mean of responses to the corresponding subscale items [42]. Non-reactance and non-judgement items were reverse coded.

##### Habit

The short form Self-Report Habit Index [46] was used to measure habit strength regarding mindless eating (i.e., eating without non-evaluative awareness and attention). Habitual engagement in mindless eating as opposed to ME was chosen because scales for assessing habitual engagement in health behaviors assume behavioral actions to occur automatically and outside of a person’s awareness. This aspect was deemed to be not applicable for the current focus on actions linked to ME. Participants were asked to indicate their agreement with the six items (e.g., “I do automatically”, “I do often”) on a five-point Likert-type rating scale from 1 (*strongly disagree*) to 5 (*strongly agree*). The scale was reverse scored, with higher values indicating less habitual mindless eating (α = 0.93).

#### ICM awareness factors

At the time of this study, no measures were available to assess belief-based constructs of ME. Questionnaire contents were based on a qualitative pilot study to determine prominent beliefs associated with ME. Items for the following ICM factors were developed by integrating the identified themes with previously published recommendations for the phrasing of social-cognitive constructs [24, 47] and previous research using the ICM [33, 37]. The following four awareness factors are treated as indices rather than scales because Cronbach’s alphas, expectedly, suggest the existence of more than one dimension of the respective ME-related actions.

##### Cognizance

Behavioral cognizance was measured using five items scored on a five-point Likert-type rating scale from 1 (*definitely false*) to 5 (*definitely true*). Items assessed the extent to which participants believed themselves to pay attention to why and what they were eating, make conscious decisions about their food, to not judge their food choices, and to not act on cravings to eat (α = 0.65).

##### Knowledge

Knowledge was assessed using seven true or false statements about ME and ME-related actions (e.g., “Mindful eating involves being aware of what I am eating” and “Mindful eating is a form of dieting that restricts certain foods such as sugar”). Answers (true/false/do not know) were dichotomized into correct (= 1) and incorrect or uncertain (= 0) answers on the respective items (α = 0.57).

##### Cues to action

Cues to action were assessed using four items rated on a five-point Likert-type rating scale from 1 (*strongly disagree*) to 5 (*strongly agree*). Participants were asked about the presence of ME-related cues in their living space (such as post-it notes or grocery lists), in the media (such as on the Internet, TV or newspaper), social environment, and internal sensations (such as stomach tightness or bloating) (α = 0.59).

##### Risk perception

Risk perception was measured using six items for perceived susceptibility and six corresponding items for perceived severity of the respective risks. Participants were asked to rate their perceived risk of different health risks (developing diabetes, high blood pressure, and gaining weight) as well as ME-related risks (overeating, giving into cravings to eat, and guilt about food choices) on a five-point Likert-type rating scale from 1 (*very low*) to 5 (*very high*). Perceived severity of these risks was assessed on a five-point rating scale from 1 (*not very serious*) to 5 (*very serious*). Mean scores were calculated separately for susceptibility (α = 0.80) and severity (α = 0.64).

#### ICM motivational factors

The following measures for motivational factors were also developed by integrating the identified themes from the qualitative pilot with published recommendations for assessing social-cognitive constructs [24, 33, 37, 47, 48].

##### Attitude

Attitudes were assessed using 19 original items corresponding to emotional and rational pros (ten items, e.g., “… help me to eat in a more healthy, balanced way.”) and cons (nine items, e.g., “… make me think too much about my food choices.”) of engaging in ME over the next month (α = 0.79). Participants were asked to indicate their agreement with the statements on a five-point Likert-type rating scale from 1 (*strongly disagree*) to 5 (*strongly agree*). Means were calculated separately for pros (α = 0.87) and cons (α = 0.67).

##### Social influence

Social influence was assessed using 18 items corresponding to subjective norms, modeling, and social support that were rated on a five-point Likert-type scale from 1 (*strongly disagree*) to 5 (*strongly agree*). Items assessed the extent to which one’s partner, best friend, friends, family, parents, and colleagues or acquaintances are perceived to (i) believe that the individual should eat mindfully (subjective norm, α = 0.95), (ii) also currently engage in ME (modeling, α = 0.85), and (iii) encourage the individual to eat mindfully (social support, α = 0.92). A “does not apply” option (= 999) was provided for participants’ responses and recoded as a blank after missing value imputation. Means were computed for cases with at least two valid values for the individual social influence factors.

##### Self-efficacy

Self-efficacy regarding ME was assessed using 19 original items in line with previous recommendations [48]. Participants were asked to rate the perceived difficulty of both sub-aspects of ME and ME in different situations on a five-point Likert-type scale from 1 (*very difficult*) to 5 (*very easy*). Sample items following the stem of “On an average day over the next month, how easy or difficult will it be for you to” include “Think about how you ate (for example, consciously enjoying a meal)?” and “Eat mindfully while around other people?”. A mean score was calculated from the corresponding scores on self-efficacy items (α = 0.90).

##### Intention

Intention to engage in ME-related actions was measured by four items that were rated on a five-point Likert-type scale from 1 (*strongly disagree*) to 5 (*strongly agree*). Items assessed participants intention over the following month to (i) obtain more information about ME, (ii) practice noticing their thoughts surrounding their eating behavior, (iii) eat at least one meal a day in a mindful manner, and (iv) to seriously attempt the latter. A mean score was computed from participants’ answers (α = 0.88).

##### Action plans

Participants were asked to indicate whether they intended to implement nine ME-related plans over the next month using a five-point Likert-type scale from 1 (*definitely not*) to 5 (*definitely yes*). Plans corresponded to ME-related actions such as practicing noticing when one is hungry or full and setting oneself reminders to eat mindfully (e.g., on a phone or through post-it notes). A mean score was computed from the corresponding answers (α = 0.78).

##### ME behavior

Engagement frequency in ME-related behavior was assessed by seven items that were rated on a five-point Likert-type rating scale from 1 (*never*) to 5 (*very often*). Participants were asked to indicate how often they engaged in different ME-related activities on an average day over the past week (e.g., “Think about why you ate (such as hunger, mealtime, food temptations)?”, “Make conscious decisions about your food?”, and “Eat mindfully while stressed?”). This abridged measure was used in addition to the comprehensive FFaMES (measuring eating-related mindfulness skills) to permit the calculation of a single score (α = 0.73) for behavioral frequency using a distinct, retroactive reference period. To allow for comparisons on ICM constructs between different levels of engagement in ME, behavior was trichotomized based on percentile cut-off points: < 2.57 (*Low ME*), 2.57 to < 3.14 (*Medium ME*), ≥ 3.14 (*High ME*).

### Data analysis

Analyses were conducted using SPSS v. 27.0 [49] considering a significance level (α) of 0.05 for two-tailed analyses. Data can be obtained from the first author upon reasonable request. Overall, missing values were low on all items (average of 0.18% per item). Little’s [50] MCAR test suggested that values were missing completely at random (χ2 (867) = 103.71, *p* = 1.00). Missing values were replaced separately for the three ME groups using expectation maximization. This method was chosen to ensure the least bias in parameter estimates and the power of subsequent analyses [51, 52]. Mahalanobis distance suggested three multivariate outliers which were removed from the subsequent analyses. Participants were classified into low (LME), medium (MME), or high (HME) engagement based on percentiles that correspond to the mean score of engagement frequency in ME. Descriptive statistics and frequencies were used to explore means and standard deviations for ICM variables, emotional eating, and facets of ME as well as percentages for categorical participant characteristics. Pre-specified multivariate analyses of variance (MANOVAs) were used to test for differences between the three groups (i.e., LME, MME, and HME) on the individual ICM items per factor. Tukey-adjusted pairwise comparisons were conducted for ICM construct means and all individual items using univariate ANOVAs. Bivariate correlations were computed to examine associations between study variables. A linear regression analysis with forward stepwise selection (*p* = 0.05) was conducted to examine model fit with the data and to identify variables uniquely associated with ME behavior. ME behavior was entered as a dependent variable and ICM constructs were entered block-wise, to examine the relative importance of predisposing factors (demographic and ME-related factors in Model 1), awareness factors (Model 2), motivation factors (Model 3), and intention (Model 4).

## Results

### Participants

As displayed in Table 1, the sample consisted of 282 participants of which 31.6% identified as male (*n* = 89), 67.7% female (*n* = 191), and < 1% outside of the binary categories (*n* = 2). The mean age of respondents was 43.21 years (*SD* = 17.19), with the majority born in Germany (95.7%, *n* = 270), currently employed (52.5%, *n* = 148), living with others (81.6%, *n* = 230), and having obtained a post-secondary degree (50.7%, *n* = 143). In total, 63.8% (*n* = 180) of the participants indicated low experience with mindfulness in general (i.e., either never having heard of or having heard of but never having attempted mindfulness). A further 26.6% (*n* = 75) reported medium levels (i.e., some proficiency with the concept and practice) and 9.6% (*n* = 27) described themselves as being (very) experienced. Overall, 91.1% (*n* = 257) indicated no dietary restrictions.

**Table 1:** Socio-Demographic Variables and Mindful Eating-Related Characteristics of Participants with LME, MME, and HME

Characteristic	Total		LME (*n* = 82)		MME (*n* = 96)		HME (*n* = 104)		*F* or χ^2^ (df)	*p *value
	M or n	SD or %	M or n	SD or %	M or n	SD or %	M or n	*SD or %*		
Age (years)	43.21	17.19	41.06	16.87	42.94	17.10	45.14	17.45	1.32 (2)	.270
*Gender*^*A*^									12.34 (4)	.015
Female	191	67.7	45	54.9	69	71.9	77	74.0		
Male	89	31.6	35	42.7	27	28.1	27	26.0		
Other	2	0.7	0	0	0	0	2	1.9		
*Education*^*A*^									4.12 (4)	.390
Low	37	13.1	8	9.8	15	15.6	14	13.5		
Medium	102	36.2	25	30.5	37	38.5	40	38.5		
High	143	50.7	49	51.2	44	45.8	50	48.0		
*Employment*^*A, B*^									3.08 (6)	.799
Employed	148	52.5	44	53.7	47	49.0	57	57.8		
Student	82	29.1	25	30.5	32	33.3	25	24.0		
Retired	35	12.4	9	11.0	11	11.5	15	14.4		
Other	15	5.3	3	3.7	6	6.3	6	5.8		
*Living Situation*									3.48 (2)	.175
Alone	52	18.4	13	15.9	14	14.6	25	24.0		
With others	230	81.6	69	84.1	82	85.4	79	76.0		
*Experience*^*A*^									47.17 (4)	< .001
Low	180	63.8	70	85.4	69	71.9	41	39.4		
Medium	75	26.6	10	12.2	21	21.9	44	42.3		
High	27	9.6	2	2.4	6	6.3	19	18.3		
*Dietary Restrictions*									1.46 (2)	.483
Yes	25	8.9	6	7.3	7	7.3	12	11.5		
No	257	91.1	76	92.7	89	92.7	92	88.5		

HME = individuals with greater engagement in mindful eating; MME = individuals with medium engagement in mindful eating; LME = individuals with lower engagement in mindful eating. Percentages are reported per mindful eating group

^A^Percentages may not add up to 100% due to rounding and missing values

^B^Multiple answers were possible (e.g., being both employed and a student)

#### Differences on demographics between levels of behavior

Frequency of ME engagement was trichotomized based on percentile cut-off points: < 2.57 (Low ME), 2.57 to < 3.14 (Medium ME), ≥ 3.14 (High ME). The sample comprised 82 individuals (29.1%) with LME (i.e., in the lowest tertile), 96 individuals with MME (34.0%), and 104 (36.9%) with HME. As displayed in Table 1, those in the highest tertile indicated significantly greater experience with mindfulness in general than those classified as MME or LME. A significantly greater proportion of women was found in MME and HME groups than in the LME group. No statistically significant differences were found between the three groups for the remaining demographic characteristics (see Table 1).

### Group differences on ICM constructs

A summary of group means and MANOVA statistics per construct are displayed in Table 2.^2^ Detailed statistics for group differences on the individual items are provided in Additional file 1: Tables 1 (FFaMES) and 2 (ICM constructs), respectively.

**Table 2:** Summary of between-group differences for LME, MME, and HME individuals per I-change model construct, emotional eating, and four facets of mindful eating (FFaMES)

Variable	Mean (*SD*)			Post-hoc comparisons ^A^	MANOVA statistics			
	LME (*n* = 82)	MME (*n* = 96)	HME (*n* = 104)		*F*	*p *value	Pillai’s trace	partial η^2^
Emotional Eating	1.87 (.76)	2.04 (.70)	1.87 (.68)	H, M, L	1.49	.180	.03	.02
Non-Reactance (FFaMES)	3.79 (.72)	3.63 (.75)	3.72 (.72)	H, M, L	1.89	.014	.12	.06
Non-Judgment (FFaMES)	3.66 (.83)	3.43 (.87)	3.60 (.90)	H, M, L	1.11	.340	.06	.03
External Awareness (FFaMES)	3.09 (.62)	3.14 (.67)	3.09 (.67)	H, M, L	.80	.648	.04	.02
Internal Awareness (FFaMES)	1.89 (.64)	2.26 (.69)	2.56 (.80)	H > M > L	4.82	< .001	.19	.10
Habit	2.51 (.89)	3.13 (.99)	3.69 (.94)	H > M > L	6.13	< .001	.24	.12
Cognizance	2.89 (.67)	3.29 (.52)	3.68 (.57)	H > M > L	13.51	< .001	.40	.20
Knowledge	.74 (.24)	.77 (.17)	.75 (.22)	H, M, L	1.40	.149	.07	.04
Cues	2.58 (.89)	2.88 (.80)	3.04 (.74)	H > M > L	6.74	< .001	.18	.09
Susceptibility	2.99 (.82)	3.05 (.77)	2.69 (.79)	H < M, L	2.82	< .001	.12	.06
Severity	3.77 (.53)	3.78 (.51)	3.78 (.53)	H, M, L	1.51	.117	.07	.03
Attitude Pros	3.54 (.64)	3.80 (.50)	3.81 (.67)	H, M > L	2.03	.005	.14	.07
Attitude Cons	3.00 (.51)	2.91 (.54)	2.65 (.56)	H < M, L	2.95	< .001	.18	.09
Subjective Norms	2.37 (1.14)	2.54 (1.13)	2.59 (1.13)	H, M, L	.80	.651	.05	.02
Modeling	2.43 (.90)	2.97 (.77)	3.27 (.79)	H > M > L	4.02	< .001	.24	.12
Social Support	2.19 (1.04)	2.69 (.97)	2.97 (.97)	H, M > L	3.54	< .001	.21	.10
Self-Efficacy	2.93 (.56)	3.20 (.51)	3.60 (.56)	H > M > L	3.85	< .001	.44	.22
Intention	2.80 (.98)	3.36 (.87)	3.55 (.88)	H, M > L	7.22	< .001	.19	.10
Planning	3.14 (.66)	3.38 (.57)	3.66 (.59)	H > M > L	2.80	< .001	.17	.09
Mindful Eating	2.04 (.30)	2.79 (.17)	3.49 (.30)	H > M > L	29.67	< .001	.87	.44

HME = individuals with greater engagement in mindful eating; MME = individuals with medium engagement in mindful eating; LME = individuals with lower engagement in mindful eating; FFaMES = Four Facet Mindful Eating Scale

^A^univariate ANOVAs and Tukey-adjusted post-hoc tests were conducted on the construct means

#### Predisposing factors

As shown in Table 2, individuals with HME reported significantly greater internal awareness (i.e., the ability to observe the effects of internal processes such as thoughts and emotions on one’s eating behavior) on the respective FFaMES subscale and less habitual mindless eating in comparison with LME and MME individuals. Those in the MME group also reported greater internal awareness and lower mindless eating habits than the LME group. No significant differences between the three groups were found for emotional eating, and the FFaMES facets of non-judgment of eating and cravings, and external awareness (i.e., the ability to observe the effects of environmental factors on one’s eating behavior). When comparing the three ME groups on their non-reactance to immediate needs to eat (FFaMES), the overall MANOVA was significant (see Table 2). However, post-hoc comparisons indicated no significant differences between the groups on the individual items (all *p*s > 0.060, see Additional file 1: Table 1).

#### Awareness factors

No significant group differences were found for knowledge about ME (see Table 2). Regarding behavioral cognizance, HME individuals indicated paying more attention to why (*F*(2, 274) = 43.99, *p* < 0.001) and how they were eating (*F*(2, 274) = 58.77, *p* < 0.001) and made more conscious decisions about their food (*F*(2, 274) = 26.67, *p* < 0.001) than MME and LME individuals. Those with MME also reported greater cognizance than LME individuals (see Table 2).

In terms of cues, those with HME were significantly more likely than those with MME and LME to have reminders for ME in their living space (*F*(2, 274) = 8.56, *p* < 0.001), to have seen information about ME in the media (*F*(2, 274) = 17.12, *p* < 0.001), to have engaged in a conversation about ME (*F*(2, 274) = 11.26, *p* < 0.001), and to perceive internal sensations as cues to engage in ME (*F*(2, 274) = 6.04, *p* = 0.003). Regarding risk perception, the groups overall significantly differed in terms of their perceived susceptibility but not in terms of the perceived severity (see Table 2). Group comparisons on individual items revealed that individuals with HME perceived the least susceptibility to the health risk of developing diabetes (*F*(2, 274) = 5.68, *p* = 0.004) and the ME-related risk of giving in to cravings to eat (*F*(2, 274) = 11.01, *p* < 0.001) in comparison to those with MME and LME.

#### Motivational factors

The groups significantly differed in terms of the perceived pros and cons associated with ME (see Table 2). Participants with HME were more likely than those with MME and LME to think that ME helps them to make healthier food choices (*F*(2, 274) = 3.52, *p* = 0.031), to prevent weight gain (*F*(2, 274) = 3.07, *p* = 0.048), to improve their health (*F*(2, 274) = 3.94, *p* = 0.021), and to feel enjoyable to them (*F*(2, 274) = 9.04, *p* < 0.001). Further, those with MME were more likely to indicate that ME is interesting to them than individuals with LME (*F*(2, 274) = 3.32, *p* = 0.038).

On the other hand, individuals with LME were more likely than those with MME and HME to indicate that ME would feel unpleasant to them (*F*(2, 274) = 5.36, *p* = 0.005), be too time consuming (*F*(2, 274) = 16.91, *p* < 0.001), stressful (*F*(2, 274) = 12.68, *p* < 0.001), useless (*F*(2, 274) = 8.68, *p* < 0.001), and would make them feel guilty about how they normally ate (*F*(2, 274) = 4.60, *p* = 0.011).

For social influence, no significant differences were found between the three groups for subjective norms (see Table 2). However, individuals with HME indicated greater engagement in ME by their partner, friends, colleagues, and family (all *p*s < 0.006), and perceived greater social support for the behavior from these individuals (all *p*s < 0.012), compared with participants in the LME and MME groups (see Additional file 1: Table 2).

Concerning self-efficacy, individuals with HME reported the highest self-efficacy for thinking about why they ate (*F*(2, 274) = 9.87, *p* < 0.001) and how they ate (*F*(2, 274) = 15.71, *p* < 0.001), making conscious decisions about food (*F*(2, 274) = 21.37, *p* < 0.001), not acting on food cravings (*F*(2, 274) = 7.12, *p* < 0.001), writing lists before shopping for groceries (*F*(2, 274) = 5.23, *p* = 0.006), planning meals (*F*(2, 274) = 15.01, *p* < 0.001) and preparing meals in advance (*F*(2, 274) = 18.20, *p* < 0.001), replacing unhealthy snacks with healthy snacks (*F*(2, 274) = 11.04, *p* < 0.001), noticing hunger and satiety cues (*F*(2, 274) = 10.67, *p* < 0.001), practicing noticing emotional triggers to eat (*F*(2, 274) = 9.11, *p* < 0.001) and external food cues (*F*(2, 274) = 7.99, *p* < 0.001) as well as setting themselves reminders to eat mindfully (*F*(2, 274) = 3.64, *p* = 0.027). Further, HME individuals indicated more ease than LME and MME individuals with eating mindfully in general (*F*(2, 274) = 47.88, *p* < 0.001) as well as while stressed (*F*(2, 274) = 18.73, *p* < 0.001), while around others (*F*(2, 274) = 21.04, *p* < 0.001), while in front of the TV or computer (*F*(2, 274) = 8.41, *p* < 0.001), and with incorporating ME-related actions into their daily routine (*F*(2, 274) = 54.34, *p* < 0.001).

#### Intention

As can be seen from Table 2, the groups significantly differed in their intention to engage in ME over the following month. Pairwise comparisons on individual items indicated that individuals with HME and MME were more likely than those with LME to intend to obtain more information about ME (*F*(2, 274) = 9.19, *p* < 0.001), and to intend (*F*(2, 274) = 20.99, *p* < 0.001) and seriously attempt to eat at least one meal per day in a mindful manner (*F*(2, 274) = 15.83, *p* < 0.001). Moreover, those with MME were more likely than individuals with LME to intend to practice noticing their thoughts surrounding their eating habits (*F*(2, 274) = 4.47, *p* = 0.012).

#### Planning

Significant differences were found between the groups regarding their action planning (see Table 2). Individuals with HME were most likely to indicate greater agreement with planning to write lists before grocery shopping (*F*(2, 274) = 4.55, *p* = 0.011), planning to plan their meals (*F*(2, 274) = 9.69, *p* < 0.001) and to prepare their meals in advance (*F*(2, 274) = 12.11, *p* < 0.001), to replace unhealthy snacks with healthy ones (*F*(2, 274) = 10.89, *p* < 0.001), to practice noticing hunger and satiety cues (*F*(2, 274) = 6.55, *p* < 0.001), to set themselves reminders to eat mindfully (*F*(2, 274) = 3.12, *p* = 0.046), and to practice noticing emotional triggers (*F*(2, 274) = 4.78, *p* = 0.009) and environmental cues to eat (*F*(2, 274) = 6.85, *p* = 0.001).

### Correlations of ICM constructs and mindful eating behavior

Cut-off points to interpret the conducted Pearson correlations were r =  ≤ 0.3 (weak correlation), r =  > 0.3 to < 0.6 (moderate correlation), and r =  ≥ 0.6 (strong correlation) [53].

Bivariate correlations indicated that the majority of ICM constructs were either weakly or moderately correlated with each other (see Table 3). Of the FFaMES facets, non-judgment, non-reactance, and external awareness were not significantly correlated with ME behavior. However, internal awareness as well as less habitual mindless eating and the awareness factors cognizance and cues to action were moderately positively associated with ME behavior. Social modeling, social support, intention, and planning were also significantly positively associated with behavior, with a moderate strength of the relationships.

**Table 3:** Bivariate Correlations for I-Change Model Constructs and Four Facets of Mindful Eating (FFaMES) (*N* = 277)

		1	2	3	4	5	6	7	8	9	10	11	12	13	14	15	16	17	18	19	20
1	Emotional Eating	–																			
2	Non-Reactance	−.70*	–																		
3	Non-Judgement	−.53*	.63*	–																	
4	External Awareness	.35*	−.46*	−.45*	–																
5	Internal Awareness	.27*	−.45*	−.39*	.35*	–															
6	Habit	−.38*	.42*	.34*	−.28*	.10	–														
7	Cognizance	−.37*	.34*	.35*	−.21*	.11	.59*	–													
8	Knowledge	.13*	−.15*	−.07	.12	.23*	−.04	.08	–												
9	Cue	−.06	−.03	−.14*	−.03	.31*	.33*	.38*	.05	–											
10	Susceptibility	.47*	−.48*	−.60*	.35*	.06	−.47*	−.45*	.08	−.01	–										
11	Severity	.25*	−.25*	−.43*	.26*	.14*	−.20*	−.06	.10	.12*	.44*	–									
12	Attitude Pros	.12*	−.18*	−.28*	.16*	.25*	−.01	.11	.27*	.24*	.28*	.41*	–								
13	Attitude Con	.18*	−.27*	−.31*	.17*	.02	−.39*	−.28*	−.03	−.04	.32*	.12*	.11	–							
14	Subjective Norms	.13*	−.27*	−.24*	.15*	.18*	−.14*	−.08	−.03	.29*	.18*	.11	.18*	.21*	–						
15	Modeling	−.14*	.01	.10	−.11	.21*	.35*	.32*	−.11	.44*	−.21*	−.11	.13*	−.09	.35*	–					
16	Support	−.02	−.15*	−.11	.02	.24*	.15*	.19*	−.05	.47*	−.02	.00	.22*	.03	.63*	.65*	–				
17	Self-Efficacy	−.37*	.40*	.42*	−.30*	.03	.66*	.64*	.01	.32*	−.52*	−.20*	.00	−.45*	−.17*	.35*	.16*	–			
18	Intention	.15*	−.22*	−.28*	.19*	.31*	.04	.14*	.15*	.23*	.15*	.31*	.57*	−.05	.31*	.15*	.32*	.10	–		
19	Planning	−.02	−.03	−.06	.07	.26*	.27*	.31*	.19*	.38*	−.01	.11	.39*	−.08	.15*	.17*	.23*	.42*	.59*	–	
20	Mindful Eating	−.04	−.04	−.07	.03	.40*	.48*	.51*	.07	.45*	−.12*	.07	.22*	−.26*	.12*	.41*	.35*	.46*	.36*	.39*	–

*p < 0.05

### Regression analysis

The corresponding statistical assumptions were met for the linear regression analysis with forward stepwise selection. Correlations between variables (all < 0.59), tolerance values (all > 0.42), and VIF (all < 2.9) indicated no threats for multicollinearity between the constructs. The histograms of standardized residuals indicated that error terms were normally distributed. The corresponding scatterplots illustrated that the data met the assumptions for linearity and homoscedasticity. The normal probability plots of standardized residuals showed that the data points closely followed the normality line.

In the final model, greater reported experience with the concept of mindfulness (β = 0.18, *p* < 0.001), more internal awareness (β = 0.20, *p* < 0.001) as well as greater behavioral cognizance (β = 0.25, *p* < 0.001), susceptibility (β = 0.14, *p* = 0.009),^3^ social support (β = 0.13, *p* = 0.006), intention (β = 0.15, *p* = 0.003), and less habitual mindless eating; β = 0.22, *p* < 0.001) were significantly positively associated with ME. The final model accounted for 54% of the variance in ME behavior (*F*(9, 248) = 32.463, *p* < 0.001).

## Discussion

This study explored how awareness-, motivational-, and action-related beliefs about eating mindfully and its domains differed between individuals with increasing engagement frequency in ME-related behaviors. Results indicated that the majority of the predisposing, awareness, and motivational factors of the ICM [27] differed between individuals with lower versus higher engagement frequency in ME-related actions.

### ICM pre-motivational factors

Individuals with HME as compared to MME and LME showed significantly greater internal awareness (i.e., the ability to observe the effects of internal processes such as thoughts and emotions on one’s eating behavior), but similar levels of non-judgment (i.e., maintaining a mental distance from one’s needs to eat), non-reactance (i.e., acceptance of one’s eating behaviors without negative self-judgment), and external awareness (i.e., the ability to observe the effects of environmental factors on one’s eating behaviors). These findings may be linked to the level of prior experience with mindfulness-based practice. Whereas a beginner to mindfulness may first only be capable of directing their present-moment attention to the eating experience, continued training may be needed to foster more complex mindfulness skills such as non-judgment and non-reactance [42, 54, 55]. In this sample, less than a fifth of HME individuals described themselves as having good or very good experience with mindfulness. This suggests that the prevalence of explicit mindfulness training, that also incorporates the attitudinal/acceptance components, may have been relatively low. Also, this may point to individuals having engaged in ME-related actions with limited knowledge of these actions being considered ME. A further reason for HME individuals only differing on internal awareness but not the remaining FFaMES subscales may be due to the participation criteria of this study. As we deliberately focused on individuals without a currently diagnosed eating disorder, these findings may be related to our sample likely having a relatively low responsiveness to external triggers to eat. This is because increased sensitivity to external food cues has previously been found in normal weight restrained eaters (i.e., individuals engaging in chronic dieting) [56–58] and among individuals with obesity-related eating behaviors [42, 59]. Future studies should consider the existing awareness levels of their population and determine which skills are thus most appropriate to train.

In terms of experience with mindfulness, HME individuals, expectedly, were more likely than MME and LME to describe themselves as more experienced. In addition, they were more aware of engaging in ME-related actions (i.e., behavioral cognizance), perceived more internal and environmental cues to action, and less habitually engaged in mindless eating compared with MME and LME. Both the internal awareness component (see FFaMES; [42]) and the awareness factors (see ICM; [27]) may be needed to prompt and repeat the engagement in ME-related actions. Such an awareness of one’s (problematic, and perhaps habitual) behaviors and actions for change are needed as a precondition for motivation and intention to engage in new health behaviors [33, 60–62]. The available external (e.g., exposure) and internal (e.g., bloating or stomach pain after overeating) cues to action may promote positive attitudes toward ME, as ME is seen as a helpful solution to reduce negative eating-related experiences. However, this mechanism seems to warrant a certain level of awareness of ME practice, one’s eating habits as well as the recognition of and ability to sit with the observed internal, bodily cues (i.e., acceptance components of mindfulness; [38]). Similarly, it is likely that a greater present-moment attention and awareness of internal triggers to eat positively affects motivational factors such as attitudes, self-efficacy, and intention to take up ME. This is because such an awareness also promotes the recognition of negative consequences of not engaging in ME on body and mind, and can in turn serve as a positive internal feedback cue when engaging in ME. Future research should investigate these mechanisms in populations with and without problematic eating behaviors to identify the most appropriate cues for each target group to prompt motivation and intention to engage in ME.

The ICM additionally suggests the importance of knowledge and risk perception in the pre-motivational phase [27]. In this study, perceived severity and knowledge were moderate and did not significantly differ between individuals with increasing engagement in ME-related actions. Although individuals may intend to (and continue to) engage in ME-related actions when perceiving a severe threat of several health risks and eating-related consequences, the general importance of risk perceptions for ME may be limited, especially when an individual is otherwise believing themselves to engage in other healthy eating practices. In terms of knowledge, the limited variance in scores may be explained by the novelty of the concept for the majority of participants. This may again be illustrated by the discrepancy in reported experience levels and behavioral frequency.

### ICM motivational factors

In terms of motivational factors, those with HME felt more positively about the benefits of ME for their health than those with less engagement. In contrast, those with LME perceived significantly greater cons of ME than HME individuals. In line with the ICM that suggests motivational factors (such as attitudes) to influence intention and behavior, our findings suggest that greater perceived cons may form a barrier to wanting to engage in ME practice. Results also point to this group potentially wanting to avoid a confrontation with their eating behaviors as they perceived ME practice as feeling unpleasant to them and as making them feel guilty about how they normally ate. The latter aspect is in stark contrast to the actual purpose of mindful eating programs, to ‘deautomatize’ eating without negative judgment of one’s food choices [38]. Therefore, it is key that researchers consider the barriers to participation of this population and utilize established, fitting methods for attitude change in their recruitment efforts (see [63, 64].

Regarding social influence, respondents with HME were more likely than those with MME and LME to perceive their social environment (i.e., friends, partner, family, acquaintances) to also engage in ME and to support them in ME. When eating mindfully is an established practice in one’s social environment, it will in turn allow an individual to learn and practice ME [65, 66]. With the increase in overweight and obesity also among the younger generations [67], it may be beneficial to conduct ME programs in social networks and settings involving, for example, both children and their caregivers.

A further motivational factor is self-efficacy [27]. Our findings indicated the greatest self-efficacy among HME individuals and the lowest among those with LME. These results are in line with prior studies that found lower levels of self-efficacy among individuals in earlier stages of behavior adoption [62, 68] Similarly, individuals with the greatest engagement in ME were more likely to have made ME-related action plans than those with MME and LME. This implies that those with lower levels of proficiency with the concept and behaviors may not be able or motivated to set appropriate goals to action. In contrast, concrete action plans may facilitate the translation from practice intention into behavior among those with firm motivation and intentions [69, 70]. Future research should investigate additional post-intentional variables such as preparatory planning and coping planning as mechanisms to bridge the intention-behavior gap for ME [70, 71].

### Implications and recommendations

In sum, future studies should assess prominent social-cognitive variables to better target participants’ beliefs, skills, support, and motivation to engage in ME practice. For this, it is recommended to make use of established theories and strategies to support the adoption of this health behavior (see [63]). Although it is too early to make definite recommendations for program components for the different target groups, we believe that individuals with prior awareness of what constitutes ME may likely benefit from programs building on existing levels of internal awareness and established healthy eating-related behaviors. In this population, program developers may want to highlight the benefits of gradual and routine practice and ensure that trainings correspond to also noticing external cues to eat as well as to the acceptance components of mindfulness (i.e., non-judgment and non-reactance). In contrast, those with limited proficiency with the concept and practice of ME may benefit from strategies that first appropriately target the knowledge of the practice, cues to action, as well as personally relevant advantages of ME and (the consequences of) habitual mindless eating. Further, interventions may want to first provide direct instructions to those individuals that describe easy-to-implement ME-related actions. This may in turn help to establish preliminary interest in attempting further ME-related trainings and change the perceived cons of the practice. Subsequently, this more tailored approach may ensure that those individuals are represented in study populations and stay on in multi-week intervention programs, and thus allow for a more accurate evaluation of the efficacy of an intervention.

### Strengths and limitations

This study is the first to examine salient beliefs about ME practice in keeping with an established theoretical model of behavior change. The results provide suggestions for future studies to better tailor program contents to increase the rates of ME practice and participants staying on. Further, results from this study contribute a better understanding of the applicability of ME in behavior change interventions for non-clinical populations. The following limitations need to be taken into account. First, this exploratory study employed a cross-sectional design. As no intervention or manipulation of study variables took place, causation of the investigated mechanisms is solely inferred from theory versus the present data. We thus recommend that future research investigates unique associations and mediated effects of the social-cognitive factors and proposed phases of the ICM using longitudinal designs and experimental research. This study presents a starting point for additional determinant research in a variety of populations. Second, the present study did not include a measure of body size. This was omitted, because the use of self-report questionnaires may have introduced measurement errors or response bias as well as body image-related discomfort [59]. As prior findings suggest a negative association of BMI and ME [72, 73], studies should investigate whether psychosocial determinants of ME differ by weight status. Third, our sample included more individuals with higher education compared with national averages. Because ME is correlated with education [74], we acknowledge that education may present an important confounder for the respective behavioral and social-cognitive measures. We therefore recommend that future studies explore determinants of ME in various demographics, and by means of direct community recruitment methods.

Fourth, a simplified index was utilized for assessing engagement frequency in ME-related actions. The complexity and variety in mindfulness-based strategies [75], the involvement of multiple different attentional processes in ME [76], and the various facets of ME [42] may have affected the accuracy with which engagement in ME was retroactively captured. Though validated ME questionnaires exist that permit the calculation of a single summary score (e.g., the Mindful Eating Questionnaire; [2]), the present study distinctly demonstrates that a separation of ME facets is necessary to accurately assess individuals’ different ME skills and the mechanisms of mindfulness strategies [77]. In this regard, the use of the validated FFaMES [42] complimented the measure for engagement frequency and additionally allowed detailed insight into participant differences on the participants’ preceding skills on attentional and acceptance facets of ME.

Lastly, we found somewhat low Cronbach’s alpha values for awareness constructs (i.e., knowledge, cues to action, cognizance, and risk perception). This was expected and suggested multiple dimensions of ME-related actions (i.e., awareness and non-evaluative components), which may have somewhat obscured the outcomes pertaining to the presented regression analysis. However, these measures were treated as indices and between-group differences were examined at item-level to gain detailed insight into the underlying belief structures.

### Conclusion

Individuals need to be treated differently when promoting ME adoption with respect to their psychosocial characteristics, rather than as a single group with homogenous baseline beliefs, abilities, support, and motivation. Individuals that engaged the least frequent in ME-related actions reported more habitual mindless eating, significantly lower experience with the concept of mindfulness as well as lower behavioral cognizance, and internal and external cues to engage in ME. Program developers promoting ME should ensure the presence of internal and external cues to engage in ME, knowledge of the practice, and behavioral cognizance before targeting motivational factors. To increase a person’s motivation to adopt ME, perceived advantages of the practice, the perceived disadvantages, the engagement and support by the individual’s social environment as well as practice self-efficacy may be relevant constructs to target. In turn, this tailoring may ensure that individuals stay on in multi-week intervention programs and successfully adopt ME practice. As this study presents a starting point for identifying determinants of ME, it seems wise to conduct further research on relevant psychosocial factors in different relevant populations (i.e., individuals with and without problematic or obesity-related eating behaviors) before ‘mindlessly’ evaluating programs with a one-size-fits-all approach. Longitudinal studies are warranted to validate the current findings and to examine changes in motivation and behavior.

## Supplementary Information

**Additional file 1**. Supplementary tables displaying the between-subject effects for individuals with lower, medium, and higher engagement in mindful eating-related actions on items of I-Change Model indices and the Four Facet Mindful Eating Scale (FFaMES).

## Data Availability

Data and materials can be obtained from the first author upon reasonable request.

## Footnotes

1 Data from individuals with more than 15% missing values was excluded (*n* = 20). Excluded individuals did not significantly differ from the remaining sample in terms of their age, gender, education, or engagement in ME.

2 Results did not significantly differ after controlling for gender. It was preferred to present the uncontrolled effects of the MANOVAs.

3 Although the correlation was negative for susceptibility, it is reversed in the regression due to a suppressor effect with cognizance.
