# Supplementary material for: Examining socio-cognitive factors and beliefs about mindful eating in healthy adults with differing practice experience: a cross-sectional study
Source: BMC Psychol. 2022 Nov 15;10:268. doi: 10.1186/s40359-022-00977-4 (PMC9664610; doi:10.1186/s40359-022-00977-4)
Supplement: Supplementary file 1 — Additional file 1. Supplementary tables displaying the between-subject effects for individuals with lower, medium, and higher engagement in mindful eating-related actions on items of I-Change Model indices and the Four Facet Mindful Eating Scale (FFaMES). [file 40359_2022_977_MOESM1_ESM.docx]

# Supplementary Table 1

Between-Subject Effects for LME (*n* = 82), MME (*n* = 96), and HME (*n* = 104) Individuals on Four Facets of Mindful Eating (FFaMES) and Emotional Eating

| Item | Mean (*SD*) | | | *F* | *p-*value | Post-hoc comparisons |
| --- | --- | --- | --- | --- | --- | --- |
|  | LME | MME | HME |  |  |  |
| Non-Reactance ^A^ |  |  |  |  |  |  |
| I feel a sense of urgency to follow my thoughts to eat. | 3.04 (1.09) | 3.09 (.94) | 2.91 (.94) | .89 | .412 | H, M, L |
| I eat to make myself feel better. | 3.07 (1.14) | 3.38 (.94) | 3.62 (1.12) | 2.30 | .102 | H, M, L |
| I use food to numb my emotions. | 4.30 (1.08) | 4.04 (1.05) | 4.30 (.90) | 2.07 | .129 | H, M, L |
| My emotions control what I eat. | 3.62 (1.12) | 3.33 (.93) | 3.50 (1.01) | 1.78 | .170 | H, M, L |
| I distract myself with food when I have uncomfortable thoughts. | 4.09 (1.07) | 3.91 (1.03) | 4.14 (.96) | 1.47 | .233 | H, M, L |
| My emotions control how much I eat. | 3.82 (1.02) | 3.46 (1.03) | 3.66 (1.04) | 2.67 | .071 | H, M, L |
| I push uncomfortable feelings away by eating. | 4.29 (.97) | 4.03 (1.01) | 4.13 (1.00) | 1.40 | .248 | H, M, L |
| I get carried away by my thoughts while I eat. | 2.82 (1.16) | 3.19 (1.03) | 3.09 (.95) | 2.84 | .060 | H, M, L |
| I escape uncomfortable emotions by eating. | 4.18 (1.01) | 4.07 (.93) | 4.16 (.97) | .33 | .717 | H, M, L |
| Non-Judgment ^A^ |  |  |  |  |  |  |
| I get frustrated when I notice a craving. | 3.82 (1.07) | 3.63 (1.04) | 3.83 (1.10) | 1.07 | .343 | H, M, L |
| I get mad at myself for overeating. | 3.44 (1.29) | 3.19 (1.23) | 3.57 (1.24) | 2.36 | .097 | H, M, L |
| I get mad at myself for eating unhealthy foods. | 3.60 (1.01) | 3.32 (1.06) | 3.49 (1.11) | 1.47 | .233 | H, M, L |
| I feel like a bad person when I give into a craving to eat. | 3.73 (1.15) | 3.42 (1.21) | 3.73 (1.10) | 2.29 | .103 | H, M, L |
| I judge my eating as good or bad. | 3.06 (1.08) | 2.84 (1.12) | 2.91 (1.15) | .85 | .429 | H, M, L |
| I feel guilty when I eat too much. | 3.61 (1.11) | 3.59 (1.37) | 3.59 (1.14) | .15 | .857 | H, M, L |
| I believe that I should not have certain thoughts about food. | 3.51 (1.36) | 3.41 (1.29) | 3.65 (1.18) | .96 | .385 | H, M, L |
| I escape uncomfortable emotions by eating. | 4.14 (1.07) | 4.02 (1.04) | 3.92 (1.19) | .87 | .420 | H, M, L |
| External Awareness |  |  |  |  |  |  |
| I notice how my desires to eat change when  I’m surrounded by my favorite foods. | 3.47 (.91) | 3.44 (.90) | 3.41 (.88) | .08 | .923 | H, M, L |
| I notice when the smell of food makes me want to eat. | 3.21 (1.02) | 3.29 (.92) | 3.33 (.91) | .21 | .812 | H, M, L |
| I notice when the sight of food makes me want to eat. | 2.99 (.91) | 3.11 (.94) | 3.20 (.87) | 1.24 | .290 | H, M, L |
| I notice how my desires to eat change when I see or smell something delicious. | 3.39 (.85) | 3.46 (.82) | 3.27 (.87) | 1.28 | .280 | H, M, L |
| I notice that I tend to eat more when others are also eating. | 2.94 (1.15) | 2.99 (1.18) | 2.84 (1.03) | .48 | .618 | H, M, L |
| I notice how talking about food affects my desires to eat. | 2.56 (.93) | 2.67 (1.08) | 2.58 (.97) | .31 | .736 | H, M, L |
| Internal Awareness |  |  |  |  |  |  |
| I pay attention to my mood while I eat. | 1.53 (.68) | 2.27 (.97) | 2.41 (1.05) | 21.77 | < .001 | H, M > L |
| I notice my thoughts after I eat. | 2.10 (1.05) | 2.50 (1.03) | 2.79 (1.08) | 9.39 | < .001 | H, M > L |
| I pay attention to how my food affects my mood. | 1.91 (.98) | 2.40 (.99) | 2.56 (1.09) | 9.28 | < .001 | H, M > L |
| I pay attention to my thoughts while I eat. | 1.68 (.91) | 2.01 (.85) | 2.48 (1.02) | 16.98 | < .001 | H > M, L |
| I notice my mood after I eat. | 2.13 (1.02) | 2.56 (.99) | 2.78 (1.00) | 9.35 | < .001 | H, M > L |
| I pay attention to how my surroundings affect my desires to eat. | 2.06 (.98) | 2.20 (.91) | 2.52 (1.01) | 5.40 | .005 | H > L |
| Emotional Eating |  |  |  |  |  |  |
| When I feel anxious, I find myself eating. | 1.61 (.88) | 1.74 (.77) | 1.61 (.70) | .90 | .407 | H, M, L |
| When I feel blue, I often overeat. | 2.06 (.96) | 2.20 (.88) | 1.89 (.87) | 2.87 | .059 | H < M |
| When I feel lonely, I console myself by eating. | 1.97 (.97) | 2.19 (.87) | 2.01 (.85) | 1.49 | .226 | H, M, L |

*Note*. HME = individuals with greater engagement in mindful eating; MME = individuals with medium engagement in mindful eating; LME = individuals with lower engagement in mindful eating. All items on the FFaMES were scored from 1 (*never*) to 5 (*very often*). Items for Emotional Eating were scored from 1 (d*efinitely false*) to 4 (*definitely true*).

^A^ Items for Non-Reactance and Non-Judgment are reverse coded.

# Supplementary Table 2

Between-Subject Effects for LME (*n* = 82), MME (*n* = 96), and HME (*n* = 104) Individuals on Items of I-Change Model Indices

| Item | Mean (*SD*) | | | *F* | *p-*value | Post-hoc comparisons |
| --- | --- | --- | --- | --- | --- | --- |
|  | LME | MME | HME |  |  |  |
| Habit (Mindless eating is something that …) ^A^ |  |  |  |  |  |  |
| I do often. | 2.27 (1.06) | 2.92 (1.07) | 3.63 (1.10) | 35.79 | < .001 | H > M > L |
| I do automatically. | 2.36 (1.13) | 3.06 (1.26) | 3.64 (1.14) | 25.83 | < .001 | H > M > L |
| I do without thinking. | 2.29 (1.13) | 3.02 (1.22) | 3.54 (1.22) | 24.40 | < .001 | H > M > L |
| Belongs to my daily routine. | 2.71 (1.16) | 3.44 (1.16) | 4.01 (1.06) | 29.55 | < .001 | H > M > L |
| Is something that is typically me. | 2.66 (1.15) | 3.34 (1.21) | 3.98 (1.27) | 28.54 | < .001 | H > M > L |
| Mindful eating is something that would cost me effort to do. | 2.63 (.92) | 3.07 (1.06) | 3.62 (1.09) | 20.62 | < .001 | H > M > L |
| Cognizance (I tend to…) |  |  |  |  |  |  |
| pay attention to why I am eating. | 2.82 (1.14) | 3.47 (.83) | 4.07 (.70) | 43.99 | < .001 | H > M > L |
| pay attention to how I am eating. | 2.53 (.97) | 3.54 (.75) | 3.94 (.91) | 58.77 | < .001 | H > M > L |
| make conscious decisions about my food. | 3.18 (1.05) | 3.51 (.85) | 4.13 (.80) | 26.67 | < .001 | H > M > L |
| not judge my food choices as good or bad. | 3.34 (1.17) | 3.28 (1.09) | 3.34 (1.20) | .07 | .930 | H, M, L |
| not act on immediate needs or cravings to eat. | 2.60 (1.17) | 2.78 (1.06) | 2.93 (1.19) | 1.91 | .150 | H, M, L |
| Knowledge ^B^ |  |  |  |  |  |  |
| Mindful eating is a form of dieting that restricts certain foods. | .70 (.46) | .56 (.50) | .52 (.50) | 3.02 | .042 | H < L |
| Mindful eating includes labeling food cravings as good or bad. | .39 (.49) | .39 (.49) | .38 (.49) | .00 | .997 | H, M, L |
| Mindful eating involves checking in with my hunger throughout the day. | .83 (.38) | .89 (.32) | .89 (.31) | .89 | .414 | H, M, L |
| Mindful eating involves being aware of what I am eating. | .95 (.22) | .99 (.10) | .95 (.21) | 1.40 | .248 | H, M, L |
| Mindful eating can be trained through self-observation and self-reflection exercises. | .88 (.32) | .93 (.26) | .90 (.30) | .49 | .615 | H, M, L |
| Mindful eating can prevent overeating. | .86 (.35) | .89 (.32) | .88 (.32) | .20 | .820 | H, M, L |
| Mindful eating can prevent weight gain. | .62 (.49) | .77 (.42) | .78 (.44) | 3.18 | .043 | H, M, L |
| Cues |  |  |  |  |  |  |
| I have things in my living space that remind me to eat mindfully. | 2.64 (1.30) | 2.75 (1.21) | 3.34 (1.26) | 8.56 | < .001 | H > M, L |
| I have seen information about mindful eating in the media. | 2.44 (1.31) | 3.15 (1.25) | 3.52 (1.15) | 17.12 | < .001 | H, M > L |
| People around me have talked to me about mindful eating. | 2.25 (1.32) | 2.46 (1.31) | 3.13 (1.38) | 11.26 | < .001 | H > M, L |
| Sensations inside of my body remind me to eat mindfully. | 3.16 (1.26) | 3.24 (1.28) | 3.71 (1.05) | 6.04 | .003 | H > M, L |
| Susceptibility (My risk of…) |  |  |  |  |  |  |
| gaining weight is. | 3.19 (1.11) | 3.26 (1.08) | 3.05 (1.11) | .98 | .378 | H, M, L |
| developing diabetes is. | 2.83 (1.17) | 2.67 (1.06) | 2.31 (1.03) | 5.68 | .004 | H < L |
| developing high blood pressure is. | 2.87 (1.22) | 2.73 (1.14) | 2.48 (1.21) | 2.54 | .081 | H, M, L |
| overeating is. | 3.05 (1.22) | 2.89 (1.16) | 2.76 (1.11) | 1.41 | .247 | H, M, L |
| giving into my cravings is. | 3.49 (1.19) | 3.66 (1.00) | 2.94 (1.18) | 11.01 | < .001 | H < M, L |
| feeling guilty about the food I ate is. | 2.69 (1.27) | 2.97 (1.26) | 2.6 (1.24) | 2.32 | .099 | H, M, L |
| Severity (To me, …) |  |  |  |  |  |  |
| gaining weight is. | 3.77 (.96) | 3.71 (.96) | 3.53 (.98) | 1.53 | .218 | H, M, L |
| developing diabetes is. | 4.66 (.55) | 4.45 (.63) | 4.49 (.68) | 2.70 | .069 | H, M, L |
| developing high blood pressure is. | 4.43 (.59) | 4.36 (.65) | 4.31 (.73) | .73 | .482 | H, M, L |
| overeating is. | 3.39 (1.11) | 3.60 (1.00) | 3.70 (.83) | 2.27 | .106 | H, M, L |
| giving into my cravings is. | 3.06 (1.07) | 3.11 (1.03) | 3.17 (1.03) | .25 | .782 | H, M, L |
| feeling guilty about the food I ate is. | 3.36 (1.10) | 3.45 (1.04) | 3.41 (1.15) | .13 | .882 | H, M, L |
| Attitude Pros (If I eat mindfully over the next month, it will …) |  |  |  |  |  |  |
| help me to make healthier food choices. | 3.68 (.86) | 3.95 (.70) | 3.98 (.88) | 3.52 | .031 | H, M > L |
| help me to prevent gaining weight. | 3.44 (1.11) | 3.77 (.93) | 3.78 (.98) | 3.07 | .048 | H > L |
| help me to manage my cravings. | 3.52 (1.08) | 3.71 (.87) | 3.65 (.97) | .84 | .434 | H, M, L |
| help me to better understand why I am eating. | 3.55 (1.06) | 3.88 (.85) | 3.77 (1.01) | 2.52 | .082 | H, M, L |
| feel enjoyable to me. | 3.32 (.92) | 3.71 (.82) | 3.89 (.94) | 9.04 | < .001 | H, M > L |
| make me feel fuller sooner. | 3.58 (.98) | 3.47 (.94) | 3.73 (.96) | 1.88 | .154 | H, M, L |
| be interesting for me. | 3.49 (1.01) | 3.83 (.76) | 3.77 (.94) | 3.32 | .038 | M > L |
| improve my digestion by eating slower. | 3.57 (1.03) | 3.71 (.93) | 3.63 (1.08) | .40 | .668 | H, M, L |
| improve my health. | 3.61 (.98) | 3.94 (.87) | 3.95 (.84) | 3.94 | .021 | H, M > L |
| help me to eat in a more healthy, balanced way. | 3.79 (.89) | 4.00 (.79) | 3.94 (.96) | 1.22 | .297 | H, M, L |
| Attitude Cons (If I eat mindfully over the next month, it will …) |  |  |  |  |  |  |
| feel unpleasant to me. | 2.61 (1.03) | 2.48 (1.07) | 2.12 (1.11) | 5.36 | .005 | H < M, L |
| prevent me from doing other activities while eating. | 3.61 (1.24) | 3.75 (1.02) | 3.64 (.99) | .41 | .661 | H, M, L |
| prevent me from eating everything I want to eat. | 3.27 (1.10) | 3.39 (1.11) | 3.27 (1.16) | .33 | .722 | H, M, L |
| be too time consuming for me. | 3.17 (1.12) | 2.88 1.10) | 2.29 (.94) | 16.91 | < .001 | H < M, L |
| be stressful for me. | 2.92 (1.06) | 2.70 (.97) | 2.20 (.97) | 12.68 | < .001 | H < M, L |
| be useless for me. | 2.36 (1.06) | 1.84 (.85) | 1.84 (.91) | 8.68 | < .001 | H, M < L |
| prevent me from snacking. | 3.01 (.98) | 3.23 (1.08) | 3.23 (1.11) | 1.16 | .316 | H, M, L |
| make me feel guilty about how I normally eat. | 2.95 (1.20) | 2.73 (1.10) | 2.45 (1.02) | 4.60 | .011 | H < L |
| make me think too much about my food choices. | 3.19 (1.12) | 3.14 (1.06) | 2.89 (1.11) | 2.00 | .137 | H, M, L |
| Subjective Norm (… thinks I should eat mindfully over the next month) |  |  |  |  |  |  |
| My partner. | 2.39 (1.28) | 2.58 (1.27) | 2.54 (1.27) | .40 | .674 | H, M, L |
| My best friend. | 2.15 (1.14) | 2.42 (1.21) | 2.58 (1.27) | 1.98 | .141 | H, M, L |
| My friends. | 2.06 (1.04) | 2.36 (1.17) | 2.50 (1.26) | 2.35 | .098 | H, M, L |
| My family. | 2.36 (1.23) | 2.57 (1.27) | 2.55 (1.25) | .50 | .607 | H, M, L |
| My parents. | 2.31 (1.26) | 2.43 (1.28) | 2.44 (1.19) | .21 | .814 | H, M, L |
| My colleagues or acquaintances. | 1.95 (1.03) | 2.24 (1.14) | 2.36 (1.20) | 2.09 | .126 | H, M, L |
| Social Modeling (… eats mindfully) |  |  |  |  |  |  |
| My partner. | 2.07 (1.26) | 2.85 (1.28) | 3.32 (1.26) | 14.71 | < .001 | H, M > L |
| My best friend. | 2.22 (1.14) | 3.00 (1.24) | 3.22 (1.14) | 11.49 | < .001 | H, M > L |
| My friends. | 2.30 (1.06) | 3.13 (.88) | 3.11 (.94) | 14.15 | < .001 | H, M > L |
| My family. | 2.37 (1.17) | 2.94 (1.16) | 3.17 (1.02) | 7.99 | < .001 | H, M > L |
| My parents. | 2.37 (1.26) | 2.73 (1.19) | 3.17 (1.08) | 7.10 | .001 | H > L |
| My colleagues or acquaintances. | 2.26 (1.10) | 2.81 (.94) | 2.77 (.97) | 5.34 | .006 | H, M > L |
| Social Support (… encourages me to eat mindfully) |  |  |  |  |  |  |
| My partner. | 2.34 (1.39) | 2.94 (1.31) | 3.17 (1.42) | 5.83 | .003 | H, M > L |
| My best friend. | 2.04 (1.16) | 2.56 (1.03) | 3.15 (1.17) | 15.17 | < .001 | H > M > L |
| My friends. | 1.91 (.96) | 2.50 (1.03) | 2.97 (1.06) | 16.31 | < .001 | H > M > L |
| My family. | 2.24 (1.25) | 2.90 (1.21) | 2.99 (1.17) | 6.71 | .002 | H, M > L |
| My parents. | 2.23 (1.25) | 2.59 (1.16) | 2.89 (1.13) | 4.88 | .009 | H > L |
| My colleagues or acquaintances. | 1.91 (1.05) | 2.31 (1.07) | 2.46 (.94) | 4.57 | .012 | H > L |
| Self-Efficacy (On an average day over the next month,  how easy or difficult will it be for you to …) |  |  |  |  |  |  |
| Think about why you ate? | 3.22 (.94) | 3.61 (.88) | 3.83 (.86) | 9.87 | < .001 | H, M > L |
| Think about how you ate? | 3.15 (.93) | 3.69 (.80) | 3.88 (.84) | 15.71 | < .001 | H, M > L |
| Make conscious decisions about your food? | 3.09 (1.05) | 3.59 (.79) | 3.95 (.81) | 21.37 | < .001 | H > M > L |
| Not feel guilty about your food choices? | 3.44 (1.20) | 3.59 (1.14) | 3.63 (1.19) | .68 | .509 | H, M, L |
| Not act on immediate needs or cravings to eat? | 2.68 (1.17) | 2.74 (.97) | 3.19 (1.07) | 7.12 | < .001 | H > M, L |
| Writing grocery lists before you go shopping? | 3.68 (1.30) | 3.88 (1.15) | 4.21 (.97) | 5.23 | .006 | H > M, L |
| Planning your meals in advance? | 3.33 (1.26) | 3.43 (1.06) | 4.11 (.98) | 15.01 | < .001 | H > M, L |
| Preparing your meals in advance? | 2.68 (1.12) | 2.89 (1.07) | 3.57 (1.00) | 18.20 | < .001 | H > M, L |
| Replacing unhealthy snacks with healthy snacks? | 3.00 (.97) | 3.03 (1.04) | 3.63 (1.12) | 11.04 | < .001 | H > M, L |
| Practicing to notice when you are hungry or full? | 3.00 (.96) | 3.21 (1.00) | 3.65 (.97) | 10.67 | < .001 | H > M, L |
| Practicing to not judge your eating behaviors as good or bad? | 3.23 (1.17) | 3.51 (1.07) | 3.53 (1.14) | 1.97 | .141 | H, M, L |
| Practicing to notice when your emotions make you want to eat? | 2.96 (.99) | 3.35 (.91) | 3.57 (.95) | 9.11 | < .001 | H, M > L |
| Practicing to notice when your environment makes you want to eat? | 3.18 (1.02) | 3.38 (.82) | 3.69 (.86) | 7.99 | < .001 | H > M, L |
| Setting yourself reminders to eat mindfully? | 2.76 (1.04) | 3.03 (1.00) | 3.19 (1.02) | 3.64 | .027 | H > L |
| Eat mindfully? | 2.56 (.87) | 3.18 (.88) | 3.84 (.86) | 47.88 | < .001 | H > M > L |
| Eat mindfully while stressed? | 2.08 (.90) | 2.36 (.95) | 2.95 (1.07) | 18.73 | < .001 | H > M, L |
| Eat mindfully while around other people? | 2.47 (1.00) | 2.98 (.97) | 3.46 (1.04) | 21.04 | < .001 | H > M > L |
| Eat mindfully while in front of the TV or computer? | 2.46 (1.17) | 2.53 (1.07) | 3.06 (1.09) | 8.41 | < .001 | H > M, L |
| Incorporate mindful eating into your daily routine? | 2.44 (.84) | 2.95 (.93) | 3.78 (.84) | 54.34 | < .001 | H > M > L |
| Intention (Over the next month, …) |  |  |  |  |  |  |
| I intend to obtain more information about mindful eating. | 2.41 (1.01) | 2.95 (1.09) | 3.06 (1.14) | 9.19 | < .001 | H, M > L |
| I intend to practice noticing my thoughts surrounding my eating habits. | 3.06 (1.09) | 3.50 (.91) | 3.34 (1.05) | 4.47 | .012 | M > L |
| I intend to eat mindfully for at least one meal each day. | 2.85 (1.09) | 3.49 (1.00) | 3.85 (1.04) | 20.99 | < .001 | H > M > L |
| I will seriously attempt to eat mindfully for at least one meal each day. | 2.89 (1.21) | 3.53 (1.08) | 3.82 (1.06) | 15.83 | < .001 | H, M > L |
| Planning (Please indicate the extent to which you intend to implement these plans over the next month.) |  |  |  |  |  |  |
| Writing grocery lists before I go shopping. | 3.62 (1.42) | 3.81 (1.26) | 4.17 (1.15 | 4.55 | .011 | H > L |
| Planning my meals in advance. | 3.59 (1.09) | 3.70 (.93) | 4.16 (.94) | 9.69 | < .001 | H > M, L |
| Preparing my meals in advance. | 2.67 (1.17) | 2.83 (1.06) | 3.44 (1.16) | 12.11 | < .001 | H > M, L |
| Replacing unhealthy snacks with healthy snacks. | 3.16 (.87) | 3.44 (.90) | 3.79 (.91) | 10.89 | < .001 | H > M, L |
| Practicing to notice when I am hungry or full. | 3.43 (1.00) | 3.73 (.80) | 3.94 (.90) | 6.55 | .002 | H > L |
| Practicing to not judge my eating behaviors as good or bad. | 3.14 (1.24) | 3.36 (1.12) | 3.50 (1.11) | 2.08 | .127 | H, M, L |
| Practicing to notice when my emotions make me want to eat. | 3.14 (1.07) | 3.47 (.91) | 3.60 (1.00) | 4.78 | .009 | H > L |
| Practicing to notice when my environment makes me want to eat. | 3.09 (1.06) | 3.40 (.98) | 3.64 (.99) | 6.85 | .001 | H > L |
| Setting myself reminders to eat mindfully. | 2.32 (1.08) | 2.71 (1.11) | 2.70 (1.16) | 3.12 | .046 | H, M > L |
| Mindful Eating (When you were eating, how often did you:) |  |  |  |  |  |  |
| Think about why you ate? | 1.81 (.81) | 2.69 (.81) | 3.23 (.99) | 57.95 | < .001 | H > M > L |
| Think about how you ate? | 1.78 (.77) | 2.77 (.76) | 3.46 (.90) | 93.24 | < .001 | H > M > L |
| Make conscious decisions about your food? | 3.08 (.89) | 3.59 (.83) | 4.29 (.65) | 54.51 | < .001 | H > M > L |
| Act on immediate needs or cravings to eat? ^A^ | 2.66 (1.17) | 2.81 (.97) | 2.92 (.99) | 1.41 | .247 | H, M, L |
| Eat mindfully? | 1.92 (.79) | 2.86 (.71) | 3.78 (.72) | 141.44 | < .001 | H > M > L |
| Eat mindfully while stressed? | 1.32 (.64) | 2.04 (.72) | 3.15 (.93) | 124.74 | < .001 | H > M > L |
| Eat mindfully while around other people? | 1.69 (.85) | 2.82 (.85) | 3.68 (.82) | 126.21 | < .001 | H > M > L |

*Note.* HME = individuals with greater engagement in mindful eating; MME = individuals with medium engagement in mindful eating; LME = individuals with lower engagement in mindful eating. Examples for the mindful eating-related actions in the individual items have been omitted for brevity.

^A^ Items have been reverse coded.

^B^ Items have been dichotomized into correct (= 1) and incorrect or uncertain answers (= 0).
